# Errata

**DOI:** 10.36660/abc.20210905

**Published:** 2021-11-01

**Arq Bras Cardiol. 2021; [online].ahead print, PP.0-0**

No Artigo Original “Treinamento de Não-Cardiologistas pode Melhorar os Resultados do Tratamento de Infarto Agudo do Miocárdio com Supra de ST”, com número de DOI: https://doi.org/10.36660/abc.20200180, publicado em ahead of print no período Arquivos Brasileiros de Cardiologia, Arq Bras Cardiol. 2021; [online].ahead print, PP.0-0, incluir os autores Bruno Mahler Mioto e Pedro Silvio Farsky e suas respectivas instituições: InCor - Instituto do Coração do Hospital das Clínicas da FMUSP, São Paulo, SP - Brasil e Secretaria da Saúde do Estado de São Paulo, São Paulo, SP - Brasil. O autor Bruno Mahler Mioto inserir após o autor João Fernando Monteiro Ferreira e o autor Pedro Silvio Farsky inserir após a autora Naide Aparecida de Oliveira.

**Edição de Janeiro de 2021, vol. 116 (1), págs. 119-126**

No Artigo Original “Ablação por Cateter de Taquicardia Atrial Focal com Ativação Precoce Próxima ao Feixe de His, a Partir da Cúspide Aórtica não Coronária“, com número de DOI: https://doi.org/10.36660/abc.20180449, publicado no periódico Arquivos Brasileiros de Cardiologia, 116(1):119-126, na página 119, corrigir o nome da autora Vera Aiello para Vera Demarchi Aiello.

