# Supplementary material for: Errata
Source: Arq Bras Cardiol. 2021 Nov 1;117(5):1060. [Article in Portuguese] doi: 10.36660/abc.20210905 (PMC8682088; doi:10.36660/abc.20210905)
Supplement: Translation

# Translation: Erratum

**Arq Bras Cardiol. 2021; [online].ahead print, PP.0-0,**

In Original Article “Training Non-Cardiologists Could Improve the Treatment Results of ST Elevation Myocardial Infarction”, with DOI number: https://doi.org/10.36660/abc.20200180, published in ahead of print in the Journal Arquivos Brasileiros de Cardiologia, Arq Bras Cardiol. 2021; [online].ahead print, PP.0-0, include the authors Bruno Mahler Mioto and Pedro Silvio Farsky and their respective institutions: InCor - Instituto do Coração do Hospital das Clínicas da FMUSP, São Paulo, SP - Brazil and Secretaria da Saúde do Estado de São Paulo, São Paulo, SP - Brazil. The author Bruno Mahler Mioto insert after the author João Fernando Monteiro Ferreira and the author Pedro Silvio Farsky insert after the author Naide Aparecida de Oliveira.

**January 2021 Issue, vol. 116 (1), pages 119-126**

In the Original Article “Catheter Ablation of Focal Atrial Tachycardia with Early Activation Close to the His-Bundle from the Non Coronary Aortic Cusp”, with DOI: https://doi.org/10.36660/abc.20180449, published in the journal Arquivos Brasileiros de Cardiologia, 116(1):119-126, in page 119, correct the author's name Vera Aiello to Vera Demarchi Aiello.
